# Supplementary material for: Cancer stem cell-derived extracellular vesicles preferentially target MHC-II–macrophages and PD1+ T cells in the tumor microenvironment
Source: PLoS One. 2023 Feb 3;18(2):e0279400. doi: 10.1371/journal.pone.0279400 (PMC9897575; doi:10.1371/journal.pone.0279400)
Supplement: S1 Fig — (A) dLNGFR:mCMV-PGK:CD63-eGFP lentivector49. (B) ALDH1A1:CD63-eGFP and SRE:CD63-eGFP lentivectors. (C) ALDH1A1:SrtA and SS-mSca-LPETGG:mCMV-PGK:CD63-eGFP lentivectors. (PDF) [file pone.0279400.s001.pdf]

Figure S1

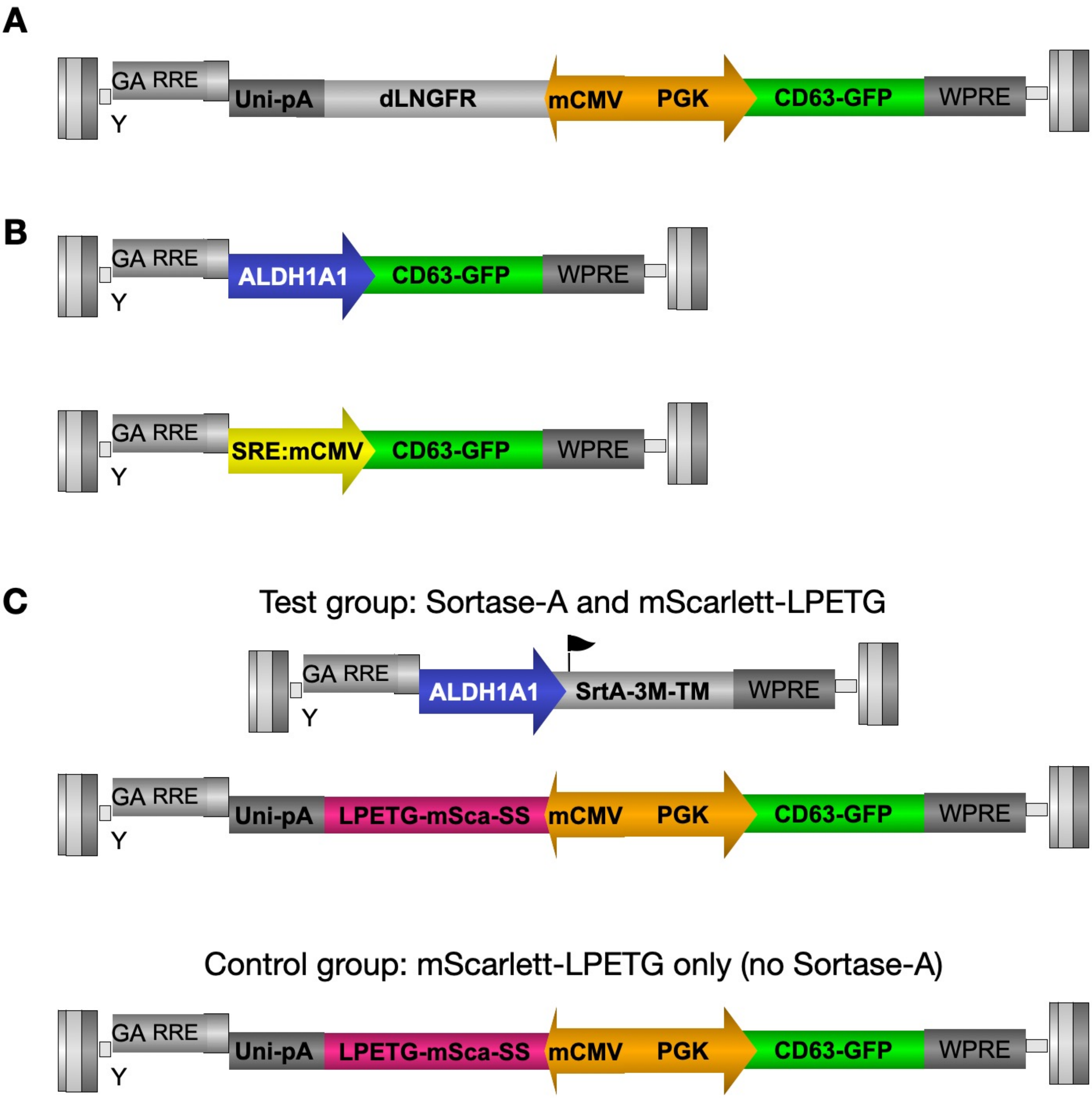
A
GA RRE
Y
Uni-pA
dLNGFR
mCMV
PGK
CD63-GFP
WPRE
B
GA RRE
Y
ALDH1A1
CD63-GFP
WPRE
GA RRE
Y
SRE:mCMV
CD63-GFP
WPRE
C
Test group: Sortase-A and mScarlett-LPETG
GA RRE
Y
ALDH1A1
SrtA-3M-TM
WPRE
GA RRE
Y
Uni-pA
LPETG-mSca-SS
mCMV
PGK
CD63-GFP
WPRE
Control group: mScarlett-LPETG only (no Sortase-A)
GA RRE
Y
Uni-pA
LPETG-mSca-SS
mCMV
PGK
CD63-GFP
WPRE
