# Supplementary figures and images for: Cancer stem cell-derived extracellular vesicles preferentially target MHC-II–macrophages and PD1+ T cells in the tumor microenvironment
Source: PLoS One. 2023 Feb 3;18(2):e0279400. doi: 10.1371/journal.pone.0279400 (PMC9897575; doi:10.1371/journal.pone.0279400)

# Figure S2

**A**

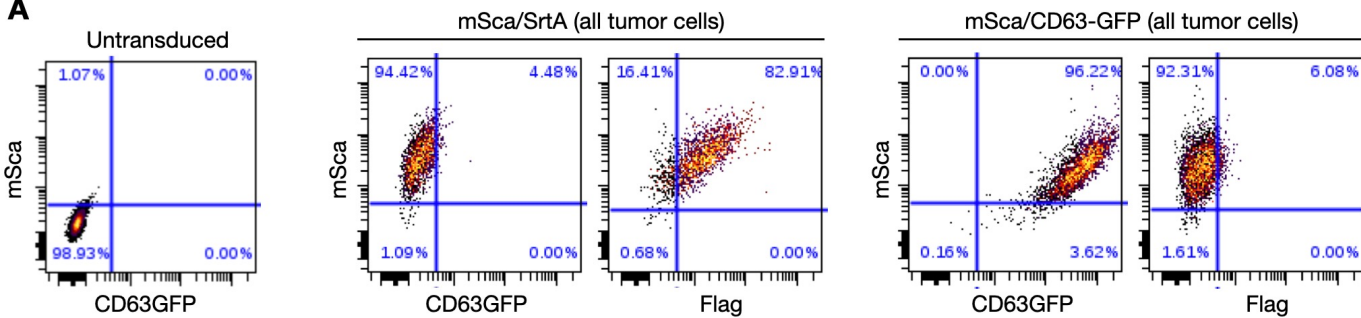

**B**

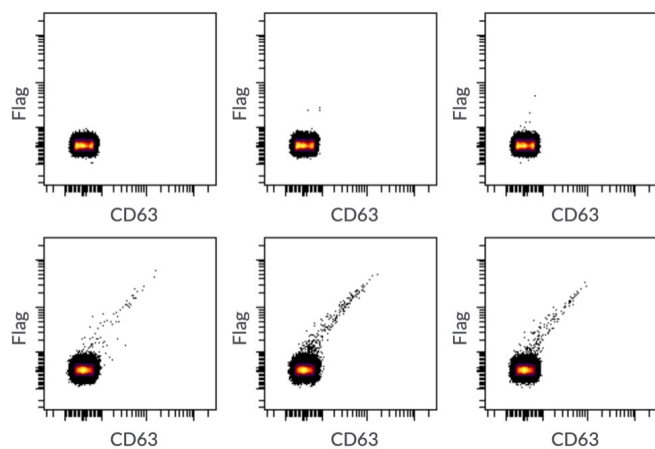

**C**

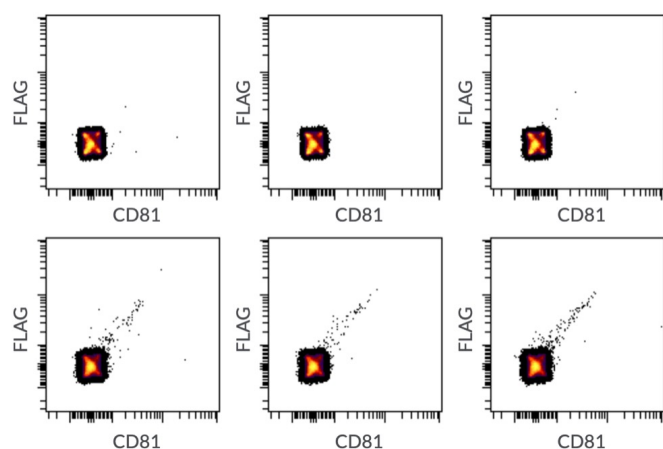

Supplement: S2 Fig — (A) mEER cells expressing mSca and SrtA (middle) or mSca and CD63-eGFP (right). Control untransduced cells are on the left. SrtA is detected by the Flag peptide. Note that these are constitutive expression vectors, not CSC specific. (B-C) Nano-flow analysis of EV preparations labeled with two fluorescent antibodies, Flag and CD63 (B) or Flag and CD81 (C). Triton treatment (top) confirms the vesicular nature of the signal. (PDF) [file pone.0279400.s002.pdf]

# Figure S3

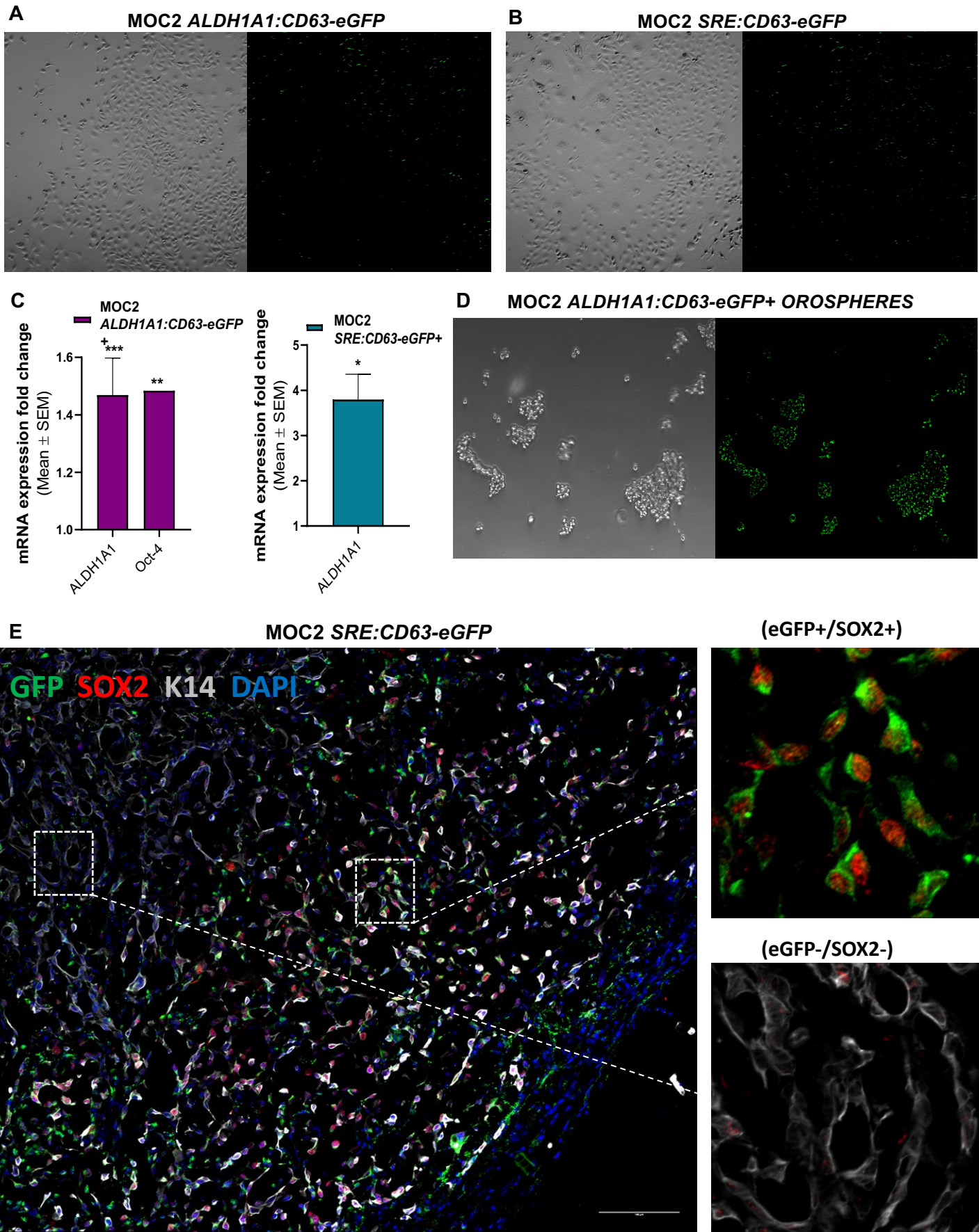

Supplement: S3 Fig — (A, B) Representative fluorescence microscopy images of cultured MOC2 ALDH1A1:CD63-eGFP cells and SRE:CD63-eGFP cells in culture. (C) Relative increase in stemness gene expression of flow sorted MOC2 eGFP + cells compared to eGFP- cells analyzed by RT-qPCR. (D) Representative images of flow sorted MOC2 ALDH1A1:CD63-eGFP+ cells growing in 3D tumorspheres specific medium. (E) Representative IF images of tumor sections presenting MOC2 SRE:CD63-eGFP tumor cells. Green cells represent the CSC population. Co-expression of CSC reporters SOX2 and eGFP are found. (PDF) [file pone.0279400.s003.pdf]

# Figure S5

A

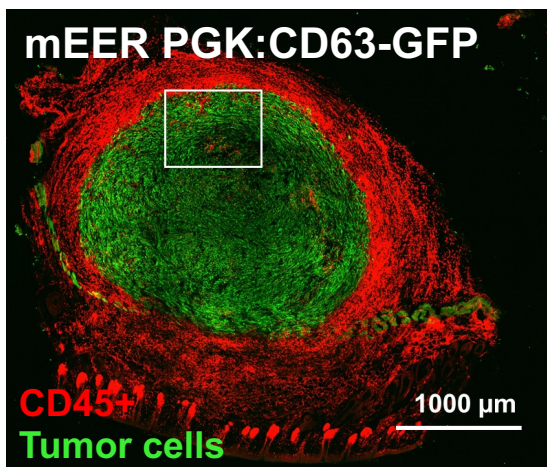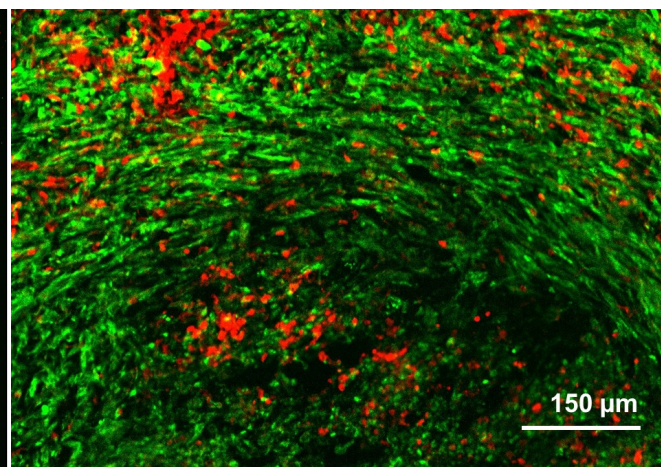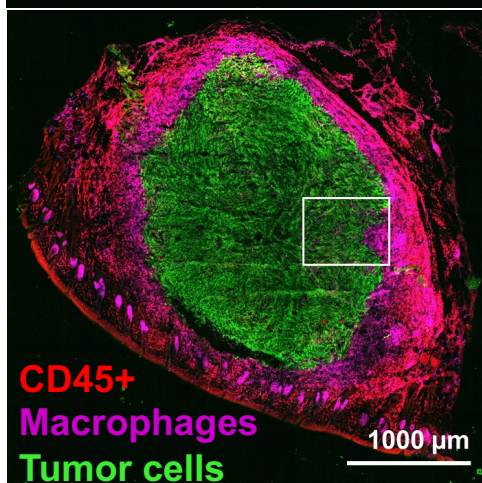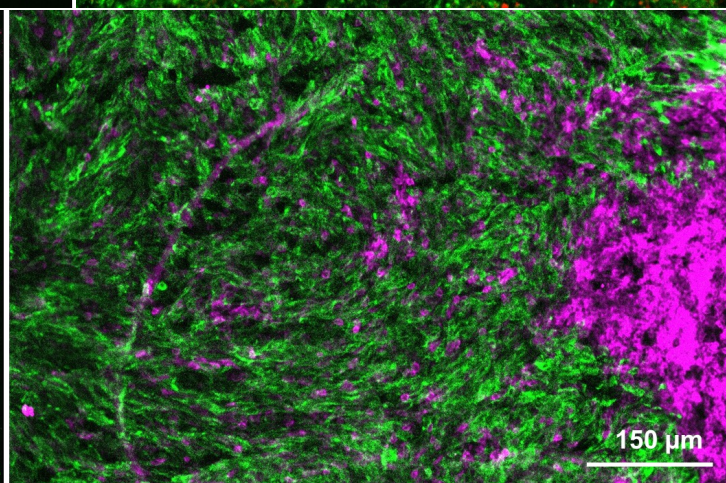

B

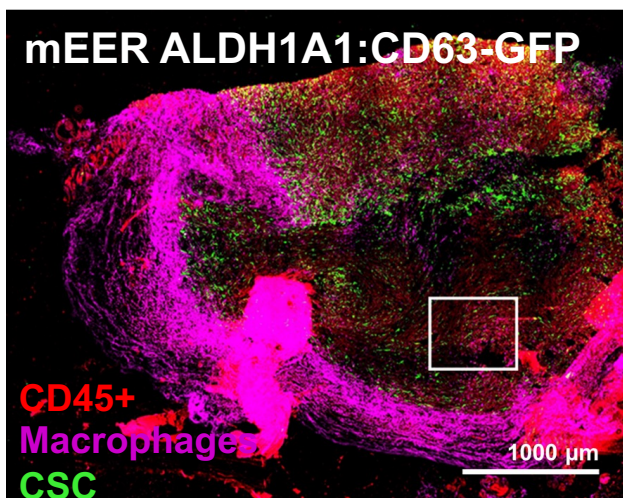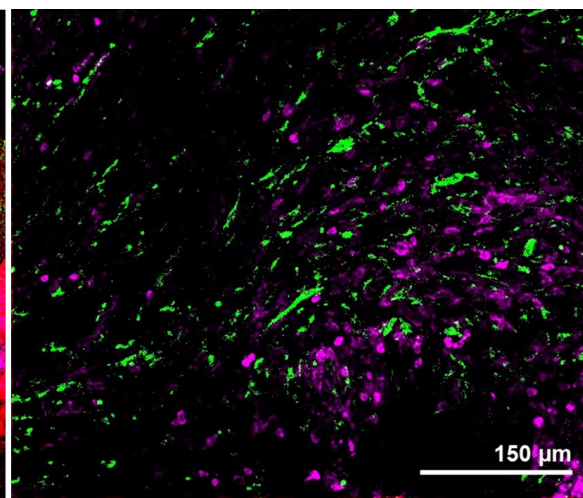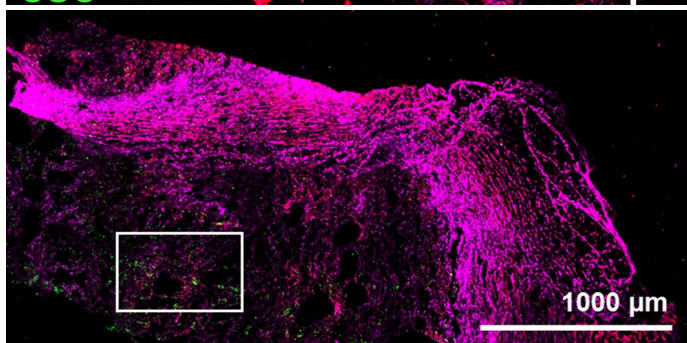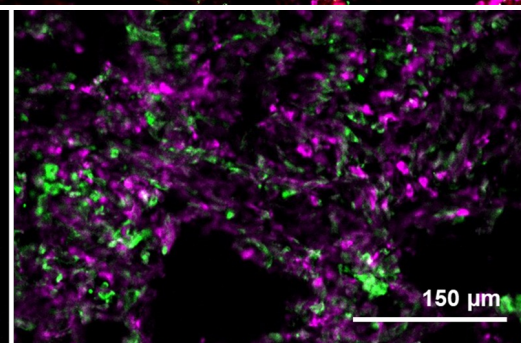

Supplement: S5 Fig — Additional representative IF images of tumor sections carrying mEER PGK:CD63-eGFP+ (A) and mEER ALDH1A1:CD63-eGFP+ (B) tumor cells. (PDF) [file pone.0279400.s005.pdf]
