# Supplementary material for: Cancer stem cell-derived extracellular vesicles preferentially target MHC-II–macrophages and PD1+ T cells in the tumor microenvironment
Source: PLoS One. 2023 Feb 3;18(2):e0279400. doi: 10.1371/journal.pone.0279400 (PMC9897575; doi:10.1371/journal.pone.0279400)

Figure S4

Gating strategy

Tumor cells

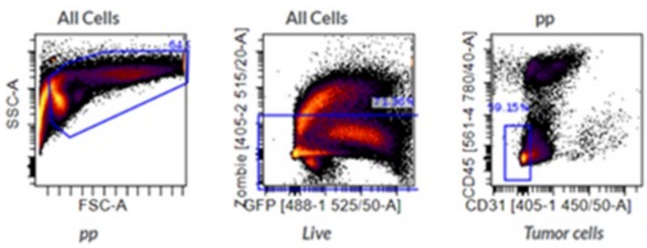

EC

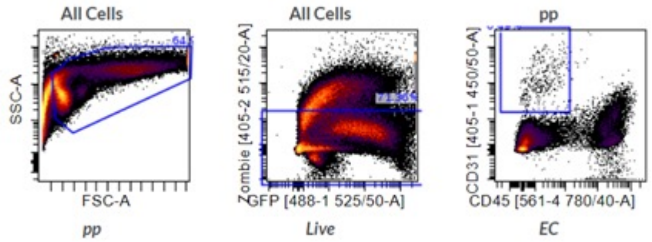

B cells

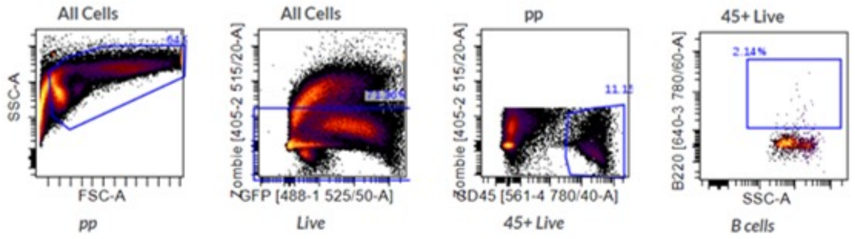

DC

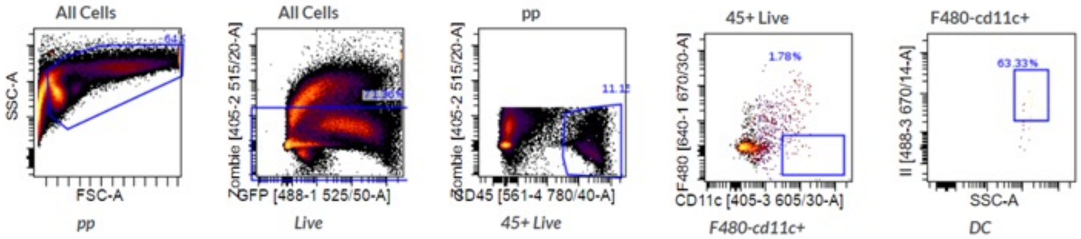

Neutrophils

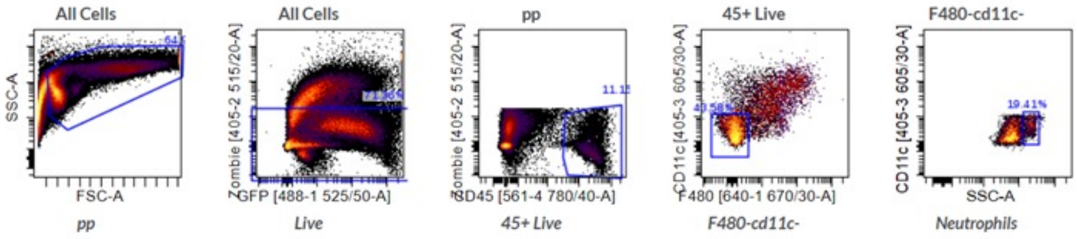

Inf Mo (11c+)

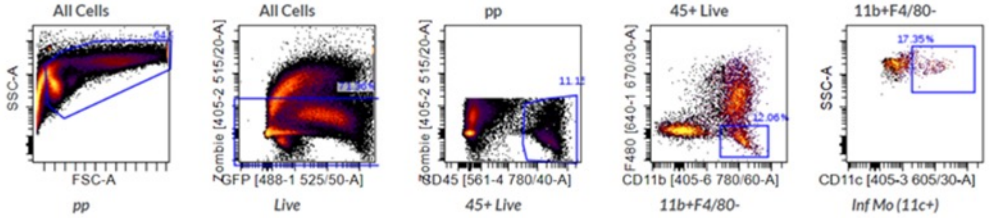

Res Mo (11c-)

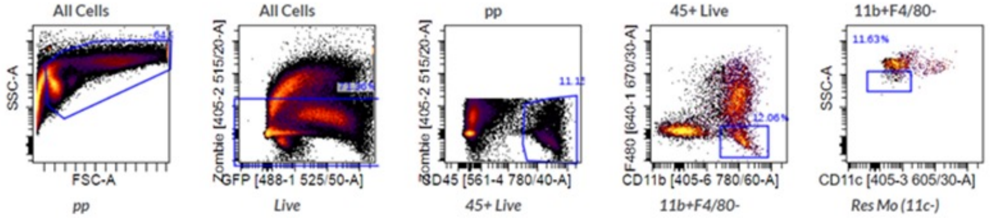

M1 Mac (II+)

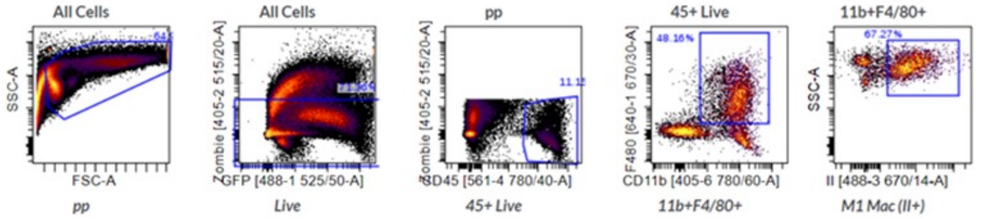

M2 Mac (II-)

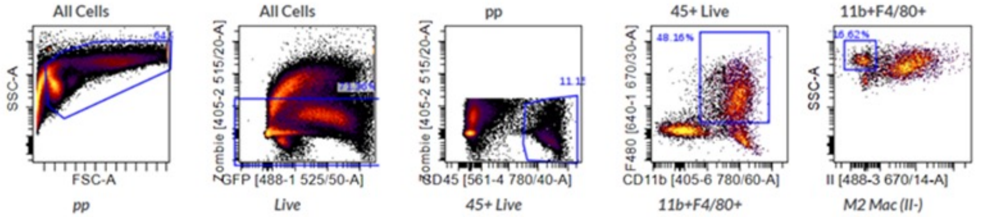

PD1+ T cells

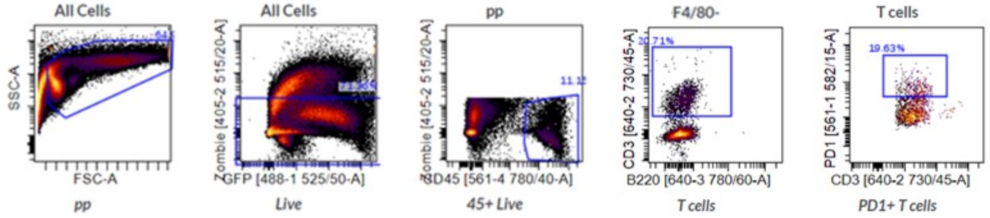

PD1- T cells

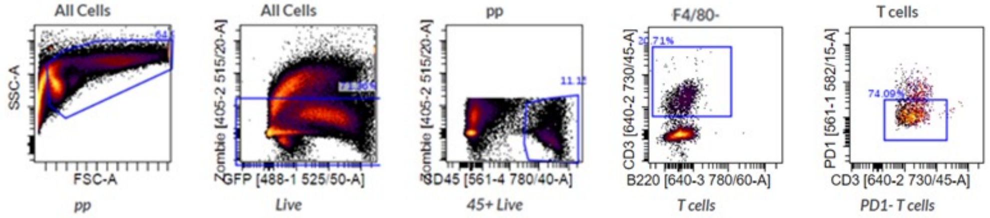

Supplement: S4 Fig — Example of the gating strategy used to characterize the immune cells subsets present in analyzed tumors of in vivo experiments using Cytobank software. (PDF) [file pone.0279400.s004.pdf]
