# Supplementary material for: Cancer stem cell-derived extracellular vesicles preferentially target MHC-II–macrophages and PD1+ T cells in the tumor microenvironment
Source: PLoS One. 2023 Feb 3;18(2):e0279400. doi: 10.1371/journal.pone.0279400 (PMC9897575; doi:10.1371/journal.pone.0279400)
Supplement: S1 Table — (DOCX) [file pone.0279400.s006.docx]

| **Gene** | **TaqMan Probe Reference** |
| --- | --- |
| **ALDH1A1** | Mm00657317_m1 |
| **Oct-4** | Mm03053917_g1 |
| **Sox-2** | Mm03053810_s1 |
| **Nanog** | Mm02019550_s1 |
| **CD133** | Mm00477121_m1 |
| **Actin** | Mm02619580_g1 |
| **GAPDH** | Mm99999915_g1 |
| **GusB** | Mm01197698_m1 |
| **Actin** | Mm02619580_g1 |
| **HPRT-1** | Mm03024075_m1 |
