# Supplementary material for: Cancer stem cell-derived extracellular vesicles preferentially target MHC-II–macrophages and PD1+ T cells in the tumor microenvironment
Source: PLoS One. 2023 Feb 3;18(2):e0279400. doi: 10.1371/journal.pone.0279400 (PMC9897575; doi:10.1371/journal.pone.0279400)
Supplement: Attachment — Submitted filename: 126756_1_rebuttal_2409020_r7x756.pdf [file pone.0279400.s007.pdf]

Full Revision

Review COMMONS

Manuscript number: RC-2022-01455
Corresponding author(s): Ferdinando, Pucci

1. General Statements [optional]

The goal of this work was to track endogenous, untouched, cancer stem cell-derived extracellular vesicles within their native microenvironment. To this end, we employed two different genetic/synthetic biology approaches that yielded fully concordant results. The Reviewers mostly understood the scope of the work but did not realize some of their requests were already addressed by our previous work. A common criticism was to improve the description of methods and experiments, and to add more schematics of expression vectors and experimental design, which we have now accomplished.

2. Point-by-point description of the revisions

Please find below our point-by-point response to reviewers' comments in blue type.

REVIEWER #1

Summary:
Provide a short summary of the findings and key conclusions (including methodology and model system(s) where appropriate).

In virtue of the classical cancer stem cells (CSC) marker ALDH1A1 and SMAD Response Element (SRE) promoter, the authors engineered CSC-derived extracellular vesicles (EVs). By performing the second sortase (SrtA)-based proximity labeling, the authors detected the immune cells that specifically interacted with the CSC-EVs and demonstrated that CSC-EVs preferentially target MHC-II- macrophages and PD-1+ T cells.

Major comments:
- Are the key conclusions convincing?
No.
CD63 is accepted as the exosome marker, but cannot represent the whole population of EVs. Especially, we do have the information on the percentage of CD63+ EVs among the total population derived by CSCs. However, it seems impossible to estimate the total population derived by CSCs. It is the inherent flaw of the strategy, which limits the accuracy of the labeling. One possible method is to label CD81+ and CD9+ EVs, together with CD63+ EVs, to study the immune cells interacting with CSC-EVs in vitro and in vivo.

We would like to thank this Reviewer for the time spent reading our manuscript and for highlighting the fact that other EVs markers could have been used to track EVs. We would like

to point out that the Sortase-A experimental strategy is independent from any assumptions on EV markers since SrtA is fused to a commonly-used transmembrane domain (from PDGFR, see Hamilton et al. Adv Biosys 2020). Nonetheless, we followed up on the Reviewer suggestion and performed additional experiments to assess the overlap between this generic membrane marker, CD63 and CD81. To this end, we have performed multicolor nano-flow cytometry and stained EVs for SrtA (via its flag peptide) and CD63 or CD81 (**new suppl. Fig. S2B-C**). We used Flag staining to detect the PDGFR transmembrane domain as a generic membrane marker (**new suppl. Fig. S1C** and ref. 50). We observed that Flag staining colocalized with both CD63+ and CD81+ EVs, indicating not only that the use of a general-purpose transmembrane domain transcends classical EV biomarkers, but also that CD63 and CD81 label largely overlapping EV subpopulations, as previously reported (Jeppesen DK *et al.* Cell 2019). Accordingly, SrtA- and CD63-GFP-based strategies yielded very similar results (Fig. 2 and 4).

Compared with the normal cancer cells, cancer stem cells are a very small population. It is reasonable to consider that the CSC-EVs is also a small population among total EVs. Therefore, it is quite questionable to compare the interaction of normal cancer cells-derived EVs and CSC-EVs with immune cells.

We fully agree with this reasoning, and it is exactly because of this contrast that our observations of the specific behavior of CSC-EVs are very relevant for CSC biology. Our experimental design includes proper control groups and is based on validated approaches (Hamilton et al. Adv Biosys 2020).

- Should the authors qualify some of their claims as preliminary or speculative, or remove them altogether?

Yes. The authors stated "such EV-mediated intercellular communication between CSC and these immune cells contributed to the observed spatial interactions and niche sharing." Not enough evidence supported the statement.

We have removed these claims from the text.

- Would additional experiments be essential to support the claims of the paper? Request additional experiments only where necessary for the paper as it is, and do not ask authors to open new lines of experimentation.

As mentioned before, if the authors could perform the labeling CD81+ and CD9+ CSC-EVs and study the interaction with immune cells, the conclusion may be more convincing.

- Are the suggested experiments realistic in terms of time and resources? It would help if you could add an estimated cost and time investment for substantial experiments.

The suggested experiments are time-consuming.

We appreciate the Reviewer acknowledging that repeating the CD63-GFP experiments in the manuscript using CD81-GFP and CD9-GFP fusion reporters is very time-consuming.

Nonetheless, we have provided proof that the SrtA approach labels both CD63+ and CD81+ EVs (**new suppl. Fig. S2B-C**), which, together with the fact that both CD63-GFP- and SrtA-based approaches yielded very similar results (Fig. 2 and 4), strongly indicates that repeating these experiments with additional reporters will add limited value to already sound conclusions.

- Are the data and the methods presented in such a way that they can be reproduced?
Yes.

- Are the experiments adequately replicated and statistical analysis adequate?
Yes.

Minor comments:
- Specific experimental issues that are easily addressable.
Yes

- Are prior studies referenced appropriately?
The references related SrtA-mediated labeling were not sufficiently referenced.

The full characterization of SrtA-based strategy was cited in reference 50 (Hamilton et al. Adv Biosys 2020). We would be happy to include any reference this Reviewer thinks is missing.

- Are the text and figures clear and accurate?
Yes

- Do you have suggestions that would help the authors improve the presentation of their data and conclusions?
No

Significance:
- Describe the nature and significance of the advance (e.g., conceptual, technical, clinical) for the field.

Cancer cells are heterogeneous. It is natural to believe that EVs are heterogeneous due to their different origin. Considering the important role of cancer stem cells during tumor development and treatment resistance acquisition, it is important to understand the function of CSC-EVs in the tumor microenvironment. However, considering the methodology is questionable, I am not sure the conclusions are convincing.
For Figure 3, there are many pieces of literature on this topic and showing the data that macrophages in CSCs niches are good for the maintenance of CSC. So, it is not novel.

We thank the Reviewer to point out the importance of understanding the function of CSC-EVs in the tumor microenvironment. We hope we have addressed the methodology issues raised by this Reviewer. Although recent students outline the relationship between CSC and

macrophages biology, very little is known about the role of EVs in this interaction. The novelty of our work stems from the use of advanced genetic engineering approaches that allow us to demonstrate directly *in vivo,* without any *in vitro* manipulation of CSC-EVs, that CSC-EVs come in contact with macrophages (and other specific immune subsets).

- Place the work in the context of the existing literature (provide references, where appropriate).

- State what audience might be interested in and influenced by the reported findings.
Cancer stem cells or extracellular vesicles are timely topics and would be interesting to people in the cancer and EV fields.

- Define your field of expertise with a few keywords to help the authors contextualize your point of view. Indicate if there are any parts of the paper that you do not have sufficient expertise to evaluate.,
EVs biology, with no special focus on CSCs.

REVIEWER #2

Summary:
In this work Dr. Pucci and colleagues use flowcytometry and in vivo approaches to define the potential role of EVs originating from cancer stem cells to mediate intercellular communication with cells of immune origin in the cancer microenvironment.
The work is interesting, the team used specific promoters to drive the expression of EV markers specifically in cancer stem cells. Another interesting approach is the use of sortase to label neighbour cells in the cancer microenvironment.

Overall the reviewer has the impression the work is quite superficial and the conclusions cannot be claimed by the results presented in the paper for the following reasons:

1. Cancer stem cells are a relatively small fraction when compared to the entire cancer cell population, therefore it is possible that the EV released tend to accumulate in macrophages because those are cells competent for specialized internalization and clearance of EV (e.g. PMID: 30745143). Second accumulation in macrophages does not mean any kind of signaling, it may just be that the EVs are degraded.

We thank the Reviewer for understanding and appreciating our work. We would like to point out that the novelty of our work is the identification of a specific macrophage subset (MHC-II– macrophages) that is mainly targeted by CSC-EVs (Fig.2). We observed a selective enrichment of these macrophage subset when tracking CSC-EVs, which argues against passive uptake, as the Reviewer seems to suggest. Moreover, these class-II negative macrophages were also found at increased frequency within the CSC niche (Fig.4), further suggesting an active process. This information is not trivial and cannot be inferred from the literature.

We are not claiming any signaling mechanism, which will be the focus of future work, including the role of CSC-EVs on maintaining MHC-II– immunosuppressive macrophage populations. We have amended the main text to clarify these points.

2. The sortase experiment is very interesting, however key controls are missing. For example, a thorough in vitro characterization of the system is needed:
a. No clear description of the vectors used is provided (how is the labelling fluorescent protein released by the cells? How far can the protein diffuse?);
b. The sortase's labelling efficiency is not characterized.
c. Which proteins are targeted by sortase in the acceptor cells? There is any protein that can specifically be labelled by sortase on the cell surface of acceptor cells? This is not explained or validated.
d. The authors claim that the labelling is provided by EVs harboring sortase on their surface, however also the plasma membrane of the cells may efficiently label cells. This should be explored and discussed. Which is the enzymatic sortase activity present on the EVs? How the authors can exclude that the red fluorescent protein is simply internalized by the neighbor cells? This should also be evaluated.

We thank the Reviewer for the interest in the SrtA-based approach, which we have thoroughly described and validated in Hamilton et al. Adv Biosys 2020 (ref. 50). We apologize if we did not reference that work properly. In that publication, we characterized SrtA labelling efficiency under various conditions and with different substrates, we mentioned which proteins can be targeted by SrtA on the surface of cells (that is, any protein with an N-terminal glycine, such as MHC-I, VE-Cadherin, CD19, integrins, …). We have clarified the above details in the new version of the manuscript.
We apologize for not including a schematic of the lentiviral vectors used, that we have now added (**new suppl. Fig. S1**). These schematics show that the red fluorescent protein is released from cells because it is fused to a signal sequence. In order to control for internalization of the red fluorescent protein by neighboring cells, we have used a control group in which CSC do not express SrtA while the bulk of tumor cells (including CSC) still secrete the red fluorescent protein. The values for SrtA activity are calculated by subtracting baseline internalization of red fluorescent protein by each individual immune subset. We have amended the Methods section to clarify this.
We are glad to hear that the Reviewer fully understood how the SrtA-based approach works. As this Reviewer mentions, it is not possible to discriminate between CSC and CSC-EVs since SrtA is present on both. This is a limitation of current EV technology in general. Although we were careful in wording our results and conclusions, we have revised the manuscript to take this into further consideration. The manuscript now claims that the SrtA approach unveils short-range interactions between CSC, CSC-EVs and immune cells due to their proximity.

3. Method section should be expanded, map of vectors provided and possibly deposited.

We apologize for not including a schematic of the lentiviral vectors used, that we have now added (**new suppl. Fig. 1**). We have expanded description of the Methods. We will promptly deposit the lentiviral transfer plasmids employed in this work with Addgene upon publication.

Minors:
The paper should be expanded, and experiments better described.

We have expanded the paper and the description of the experiments, as requested.

CROSS-CONSULTATION COMMENTS
I find the comments of Reviewer 1 important to be addressed. I might have under-estimated the amount of time necessary to revise the work.
I still believe the material and method section is insufficient.

We thank the Reviewer for acknowledging that major revision of our work is very time-consuming. We hope to have addressed both Reviewer 1 and 2 comments.

Significance:
The paper present technological innovation that can result of interest for the large audience of EV enthusiasts. The scientific advancement is limited since the conclusion: These results suggest that combination therapies targeting CSC, tumor macrophages and PD1 may synergize, is known and the work presented does not really support it since there is no evidence the EVs have any signaling role. Perhaps the authors should work more on tightening their result to a cell biology perspective of cancer niche interaction.

We thank the Reviewer for understanding the technological innovation. We will remove the statement on combination therapy and refocus on a cell biology perspective.

REVIEWER #3

In this paper, the authors used genetically engineered CSC-derived EVs to perform sortase-mediated in vivo proximity labeling and interrogate interactions of these vesicles with immune cells in the TME. The authors show that these EVs mediate intra-tumoral recruitment of immune cells, MHC class II(-) macrophages and PD1+ T cells, to the CSC niche and define EV-mediated special interactions of these immune cells within this niche.
The manuscript is timely and novel, as it introduces a new experimental platform for identification, characterization and monitoring of CSC-derived EVs within the TME. Much has been recently learned about tumor cell-derived "tEVs", while almost nothing is known about CSC-derived tEVsCSC. Here, using genetic engineering, the authors have created specifically labeled fluorescent (GFP) tEVsCSC and studied interactions of these vesicles with immune cells in the TME. Two different HNSCC mouse models, MOC2 (carcinogenesis-dependent) and mEER (Ras dependent), were used. CSC populations were identified as cells with the brightest GFP fluorescence (~5%). These cells also expressed the known stem cell markers and formed

oospheres in vitro. The authors then show that tEVcsc preferentially targeted MHC II (-) macrophages, which avidly uptake these EVs. tEVcsc also showed preferential tropism towards PD1+ T cells. Further, the authors demonstrate that location-dependent labeling indicates the presence and "clustering" of MHC II (-) macrophages and PD1+ T cells in the same niche within the TME.
The generation of genetically modified labeled fluorescent tEVcsc and tEVs and in vitro as well as in vivo analyses of their interactions with immune cells in the TME were technically demanding. These studies were expertly performed, and the results are convincing. The data presentation is adequate, but the figure legends are sparce, and the text is densely narrated and somewhat difficult to read. Some more clarity in Results and more explicitly documented correlative data would clarify and enhance the message the authors convey.

We thank the Reviewer for fully understanding the novelty of our work. We agree that the description of results and figures can be improved. We have now clarified the narration of results and figure legends so they can be better understood.

The Discussion is rationally written, but the comments in Abstract and in conclusions about combination therapies and targeting CSC, tumor macrophages and PD1 to lower HNSCC recurrence are not appropriate. There is nothing in this manuscript about immunotherapy and these comments should be deleted.

We agree with the Reviewer and we have removed these statements from the text.

Overall, this is an interesting, timely and novel manuscript using genetically modified, fluorescently labeled EVs to explore their interactions with immune cells in the TME of HNSS. Technical and experimental approaches are complex, but appear to be well done, providing an experimental model for probing cellular interactions in the TME at a single-cell level. I recommend acceptance after modifications as suggested above

Significance:
Significance is high, as it advances our understanding of the interactive role of CSC-derived EVs with macrophages and T cells in the tumor microenvironment.

We thank the Reviewer for appreciating the significance of our work.
