# Supplementary material for: Cancer stem cell-derived extracellular vesicles preferentially target MHC-II–macrophages and PD1+ T cells in the tumor microenvironment
Source: PLoS One. 2023 Feb 3;18(2):e0279400. doi: 10.1371/journal.pone.0279400 (PMC9897575; doi:10.1371/journal.pone.0279400)
Supplement: Attachment — Submitted filename: Response to Reviewers280922.docx [file pone.0279400.s008.docx]

We would like to thank the Editor and the Reviewer for taking the time to read our work and to appreciate its value to the CSC and EV community. Please find below in navy font a point-by-point response to their comments.

**Editor:**

Figure 4Aii, immune cells are depicted in the schematic presentation, but not actually in the cartoon presented. Please double check.

We agree that the schematic was misleading by not showing unlabeled immune cells (purple), that we now added along with labeled immune cells (red) within the CSC nice and immune cells labeled by total tumor cells EVs (green).

**Reviewer:**

The definition of CSCs by single or two markers remains controversial. Are ALDH1A+ or SRE positive cells accountable for the "cancer sternness" in HNSCC, or simply reflecting a specific and small fractions of heterogenous CSCs? That being said, indicating the orosphere forming efficiency of sorted ALDH1A1:CD63-eGFP+ mEER cells (Fig 1G) can partially answer the question.

We appreciate the Reviewer’s request to add the orosphere forming efficiency, which is an important parameter for relating our work to others in the literature. We have now added the assessed orosphere forming efficiency (Fig. 1G), which is 0.66%.

The measurement of the distance between tumor cell-TMA in Fig 3 should be further clarified. How many samples were included in the experiments, and whether the assay was conduced in a double-blind manner?

Thank you for noticing this missing detail in the methods. We have added that the distance measurement was made blindly by 3 independent observers. This distance was quantitatively assessed in 5 random tumor sections per mice group. These details have now been included in the methods section. Representative images were displayed in Figure 3.

In addition, are ALDA1A1+ cells in the tumors derived from total tumor population (Fig 3A) closer to TAMs? A co-staining method can help to address this. This should be important since the interactions could be altered within a heterogenous intratumoral environment.

We appreciate this Reviewer’s knowledge of the controversial issues present in the field of cancer stemness. From the Reviewer’s comment, we understand that the concern is whether our reporters label the majority the heterogeneous CSC populations or a specific subset. We have now performed the suggested experiment and co-stained with Sox2, a major CSC reporter in squamous carcinoma (PMID: 27634934). We observed a major co-localization between our reporters and Sox2 (Fig. S3E), which indicates that we effectively tagged heterogeneous CSC subpopulations. These results, the orosphere formation efficiency (Fig. 1G) and the fact that the ALDH1A1:eGFP+ and SRE:eGFP+ CSC populations express other stemness markers (Fig. 1 and S3), together indicate that our conclusions may be broadly applicable to most CSC subsets.

It should be clarified whether the attraction of TAMs or other immune cells are directly caused by EVs. More specifically, does neutralizing CD63 by antibody block the recruitment of TMAs to the peripheral or intratumoral areas?

We agree with the Reviewer that understanding the mechanism of immune cell recruitment into the CSC niche is of interest. However, this goes beyond the scope of the manuscript under consideration, which is mainly focused on the characterization of the composition of the CSC niche *in vivo*. In addition, anti-CD63 antibodies have not been shown to deplete EVs, and likely will have the opposite effect on macrophages since opsonizing EVs with antibodies will increase their binding to macrophage Fc receptors. We hope the Reviewer will understand that answering this important question will be the focus of future work.
